# Artificial Intelligence for Cancer Detection—A Bibliometric Analysis and Avenues for Future Research

**DOI:** 10.3390/curroncol30020125

**Published:** 2023-01-29

**Authors:** Erik Karger, Marko Kureljusic

**Affiliations:** 1 Information Systems and Strategic IT Management, University of Duisburg-Essen, 45141 Essen, Germany; 2 International Accounting, University of Duisburg-Essen, 45141 Essen, Germany

**Keywords:** cancer detection, artificial intelligence, machine learning, deep learning, bibliometric study

## Abstract

After cardiovascular diseases, cancer is responsible for the most deaths worldwide. Detecting a cancer disease early improves the chances for healing significantly. One group of technologies that is increasingly applied for detecting cancer is artificial intelligence. Artificial intelligence has great potential to support clinicians and medical practitioners as it allows for the early detection of carcinomas. During recent years, research on artificial intelligence for cancer detection grew a lot. Within this article, we conducted a bibliometric study of the existing research dealing with the application of artificial intelligence in cancer detection. We analyzed 6450 articles on that topic that were published between 1986 and 2022. By doing so, we were able to give an overview of this research field, including its key topics, relevant outlets, institutions, and articles. Based on our findings, we developed a future research agenda that can help to advance research on artificial intelligence for cancer detection. In summary, our study is intended to serve as a platform and foundation for researchers that are interested in the potential of artificial intelligence for detecting cancer.

## 1. Introduction

Living cells are the basic elements of all plants and animals. These cells constantly divide to replace destroyed cells or to enable the individual to grow. Although this is usually a balanced and controlled process, this genetic control can be damaged, possibly resulting in cancer [1]. Cancer is a disease that can affect most cell-based life. It befalls mankind as long as it has existed and was already recognized and acknowledged by the ancient Egyptians [2]. After cardiovascular diseases, cancer is responsible for the most deaths worldwide [3]. In 2018, there were more than 18 million new estimated cancer cases and 9.6 million cancer deaths worldwide [4]. Given the threat that cancer constitutes, researchers have already tried to understand for a long time how to cure this group of diseases in the best way.

Apart from treatments once cancer occurs, it is important to recognize the disease as soon as possible to increase the chances of recovery [5,6,7,8]. One reason why lung cancer is the deadliest cancer type is that it is difficult to detect in early stages and hard to cure in an advanced stage [9,10]. Given the high benefits of detecting cancer in early stages, new approaches are steadily being developed to support an early cancer diagnosis. Mammography was introduced in 1960 [11] and is nowadays one of the most common tools to detect breast cancer [12]. With digitalization and advances in computing power, computers have been increasingly used to support clinical practitioners with making a medical diagnosis. Computer systems that help with the detection of cancer (computer-aided detection, CAD) are an opportunity to support radiologists to achieve better detection performance [13].

One technology that receives increasing attention in recent years is artificial intelligence (AI). AI is a broad term that covers many different technologies and developments, such as machine learning (ML) or deep learning (DL) [14]. In recent years, AI has been applied in medicine for several purposes, for example, to support medical practitioners with their decision-making [15]. In the context of oncology, AI is increasingly investigated and used for several different purposes [14]. One promising application is the detection and diagnosis of cancer. Due to its potential to effectively screen or diagnose cancer or polyps [14,16], AI might be a gamechanger in the early detection of cancer diseases and is the next step in the evolution of CAD.

Not only in clinical practice but also as a research field, AI for cancer detection and diagnosis grew rapidly over the past years. Since the 2010s, the annual research on AI-supported cancer diagnosis has been steadily increasing. It is nowadays a research field with contributions from different fields, such as medicine, computer science, mathematics, and engineering. Despite the fact that there are many reviews about AI on cancer [17,18,19], there is no comprehensive study that aims to give an overview of the research field of AI in cancer detection as a whole. This is surprising, since due to the wealth of research and publications, AI for cancer detection is nowadays a huge field that is hard to oversee. This makes it difficult for interested researchers and practitioners to obtain an impression of this field, its key publications, and the main topics addressed. Given that, we aim to close this research gap by giving an overview of the literature on AI for cancer detection. The first research question we aim to address is follows:

RQ1: What are the key topics of research on AI-supported cancer detection, and what are the most contributing research constituents and articles? 

To answer our research question, we conduct a bibliometric study. A bibliometric study is a quantitative and statistical analysis of literature and allows for analyzing much larger bibliographic datasets than systematic literature reviews that follow a qualitative approach [20]. Due to their benefits, bibliometric studies have gained in popularity in recent years. Bibliometric approaches have been used in many different areas and disciplines, including pharmacy [21,22,23], oncology [24], or business and management [25]. By collecting and analyzing prior research, a bibliometric study can help to advance a field by systematically summarizing existing results. By doing so, reviews of the existing literature can also help to outline promising future research avenues and thus serve as a platform for interested scholars [26]. We follow this assumption and aim to derive future research avenues from our findings. Hence, our second research question is as follows:

RQ2: What are promising future research avenues that can help to advance the research on AI-based cancer detection?

The remainder of this article is structured as follows. In the next section, we will give an overview of AI and some foundational key terms. With that, we aim to equip readers that are not familiar with AI with basic knowledge and foundations about that technology. After that, we will explain our bibliometric approach in the third section. The bibliometric approach is divided into two phases, data collection and data analysis. Both phases are explained in more detail in two different subsections. In the fourth section, we will present the results of the bibliometric study. This is followed by a future research agenda in the fifth section. Finally, the sixth section consists of a discussion of this study’s limitations and implications, while the seventh section contains concluding remarks.

## 2. Foundations of Artificial Intelligence

The beginning of AI can be dated to the year 1943 [27] when the first concept of an artificial neuron was proposed by [28]. Thirteen years later, at the Darthmouth Conference, the term artificial intelligence was used for the first time [29]. As such, AI is one of the newest fields that is investigated in science and engineering [29] and is nowadays a complex and thriving field with numerous research topics and many use-cases and applications for companies and in practice [30,31,32]. Especially in recent years, AI has experienced extensive growth and is viewed with interest from society and practice. The main reasons are advances in computing power and increasingly more data that are available to train AI systems [33]. It is important to note that AI is a multidisciplinary field, however, that is investigated in several research fields and disciplines, including neuroscience, psychology, computer science, and mathematics [34,35].

AI is an umbrella term that comprises a lot of different algorithms and technologies. One of the most frequently used AI technologies are artificial neural networks. If artificial neural networks are multilayered and consist of several hidden layers, they are also referred to as deep learning [30,31]. Artificial neural networks aim to simulate how humans and other biological organisms learn [36]. As such, artificial neural networks are inspired by the brains of living organisms and consist of processing units, called neurons, that are connected to each other [31]. These neurons receive inputs, which then are processed according to specific rules, resulting in an output of the neuron. Often, these neurons are arranged in different modules or layers. In this context, the term deep learning describes different types of complex neural networks that consist of a large number of neurons and layers. There are several other technologies that belong to AI, such as random forests [37,38] or support vector machines [39,40]. The explanation of these technologies, however, would go beyond the scope of this paper and is not necessary to understand the further results of this study.

Although modern AI systems have a lot of capabilities, they are not intelligent in the narrow sense. To describe the capabilities of AI, [41] was the first to differentiate between two forms of AI, namely strong and weak AI. Weak AI systems are only developed for single tasks and are not generally intelligent. Additionally, they lack other human characteristics like emotions, feelings, or a conscious mind [34,41]. Although weak AI systems often seem like they would be intelligent, they only behave like that [29,42]. In contrast, strong AI, also called artificial general intelligence (AGI), describes AI systems that have the intelligence or capabilities of humans [43,44]. This not only includes the intelligence but can also mean that these systems have emotions or feelings [34]. All of today’s AI system belong to weak AI, while strong AI is not yet realized [45]. There are many assumptions about the time when a strong AI will be realized, with some researchers arguing that a strong AI might be never achieved [46]. 

## 3. Method

In this section, we explain our bibliometric approach. The conduction of a bibliometric study can be roughly divided into two steps. First, the data to be analyzed have to be collected. This step is described in the first subsection. The step of data collection is followed by the actual analysis of the data. This process is outlined in the second subsection.

### 3.1. Collection of Data

The first step was to collect the bibliometric data for our analysis. For the collection of bibliometric data, several databases exist, nowadays, with Scopus and Web of Science being among the most popular [47,48]. These databases differ in terms of their features and functionalities [49]. We decided to follow the recommendation of [20] to collect bibliometric data only from one database. We chose Scopus as the scientific database for our data collection. Scopus is a well-known database that has been used by several other bibliometric studies in the past [21,47,48,50,51,52]. Additionally, Scopus covers more journals than Web of Science and was therefore found to be suitable to identify as much research as possible [26]. Although there are other databases like Google Scholar and PubMed, we decided not to use these databases. First, Scopus has the option to develop a detailed search string and automatically download all bibliometric metadata, which is not possible with Google Scholar. Second, in comparison to PubMed, Scopus covers much more interdisciplinary research. As AI-based cancer detection is a multidisciplinary research topic, we found Scopus to be the most suitable database for conducting a bibliometric analysis. 

For the creation of our search string, we oriented ourselves to other recent bibliometric studies that investigated AI within medicine [53] and pharmacy [21]. Our search string consists of two parts, one that covers the technical terms and another that consists of the application domain. The technical part consists of general technical terms like “artificial intelligence” or “machine learning”. To search more broadly, we additionally searched for specific technologies, such as “artificial neural network”, “deep learn*”, fuzzy expert system”, or “evolutionary computation”. The applicational terms consisted of “cancer detect* and “cancer diagnos*”. The use of * symbol is due to the syntax of Scopus and allows to search for all possible word endings of the search term. This led to the following search string that was applied: 

((“artificial intelligence” OR “machine intelligence” OR “artificial neural network*” OR “machine learn*” OR “deep learn*” OR “thinking computer system” OR “fuzzy expert system*” OR “evolutionary computation” OR “hybrid intelligent system*”) AND (“cancer detect*” OR “cancer diagnos*”)).

**Figure 1:**
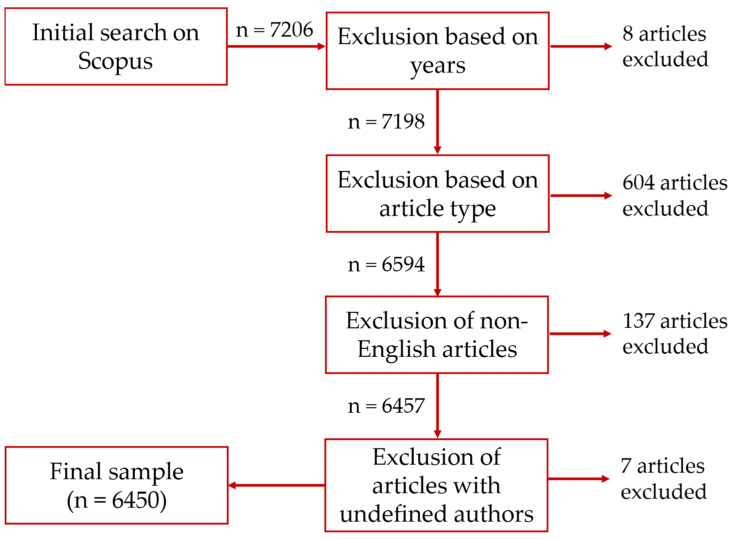
Overview of the literature collection and the exclusion criteria.

The search was conducted in title, abstracts, and keywords on 23 September 2022. The initial results consisted of 7206 documents. We did several exclusion steps to refine the data collection and to come to our final sample. First, we limited our search to 2022 as the latest year of publication. This led to an elimination of eight articles. After that, we eliminated articles based on their document type. Herein, the only documents that remained were journal articles, conference papers, or reviews. This step led to the elimination of 604 publications, with 6594 articles remaining. After that, we excluded 137 non-English articles. As a last step, we eliminated seven articles with undefined authors. In summary, this led to an elimination of 756 publications, leaving a final sample of 6450 publications. Figure 1 shows an overview of the research process, the applied exclusion criteria, and the respective numbers of eliminated publications.

### 3.2. Data Analysis

In recent years, many tools that can help to analyze bibliometric data appeared [20]. In our study, we used two tools in combination, namely Bibliometrix/Bilioshiny and VOSviewer. First, Bibliometrix is an open source R package developed by [54]. It allows for a broad variety of different forms of analysis on bibliometric data [49]. We additionally complemented Bibliometrix with Biblioshiny. Biblioshiny enables the better creation of visualizations of bibliometric data [49]. We additionally complemented Biblioshiny and Bibliometrix with VOSviewer. VOSviewer is a tool for the visualization of bibliometric data. It was developed at Leiden University in the Netherlands by the Centre for Science and Technology Studies [49,55]. VOSviewer was applied in several bibliometric studies and enables the construction of bibliometric networks that show relationships between, among others, publications, outlets, keywords, or researchers. Additionally, VOSviewer supports the creation of co-citation, bibliographic coupling, and co-authorship analysis [49,55]. Although Biblioshiny stands out in terms of statistical functionalities, we found VOSviewer a suitable tool to visualize keyword co-occurrences.

## 4. Findings

The following three sections contain the results of our bibliometric analysis. First, we will give a general overview of the sample we collected and show of the fundamental key metrics. After that, we will show the results of our performance analysis. This first contains an overview of the sources with the most publications dealing with AI for cancer detection. Second, we present the most contributing countries, funding sponsors, and affilications. After the performance analysis, we present a thematic analysis of the most relevant topics and key themes.

### 4.1. General Metrics and Overview

In this first subsection, we will present an overview of our sample and present some general metrics, such as annual production, document types, and information about the contributing authors. Table 1 shows an overview of the basic metrics of our final sample. In total, the sample consists of 6450 unique documents. These documents have been authored and co-authored by 23,854 different scholars, which is equal to 0.270 documents per author. In total, 247,762 references were cited and 9321 author’s keywords appear. Additionally, 21,192 keywords plus were identified. The 6450 documents were published in 2018 different sources and received 19.87 citations on average. Of the 6232 multi-authored articles, around 25% were developed with an international team. The timeliness of this research topic is underpinned by the fact that the average document age is only 3.72 years old. This indicates that the majority of research has been published in the last 4 years.

We compared our bibliometric data with other bibliometric studies on different topics (for an overview, see Table 2). First, it is striking that a comparatively small number of publications on AI for cancer detection have been single-author documents. Only 218 of the 6450 documents were single-authored articles, which is equal to 3.38%. This might be an indicator of the very high complexity of this topic that makes it necessary to work together in large author teams. This assumption is further underpinned by the high collaboration index for our study. The collaboration index is often used to measure the cooperation between researchers and is calculated by dividing the total number of authors that contributed to multi-authored documents by the total number of multi-authored articles [56,57]. The number of documents per author is the lowest compared to the other bibliometric studies. This shows that a lot of different researchers contribute to the field of AI for cancer detection and that this field is not dominated by only a few researchers.

**Figure 2:**
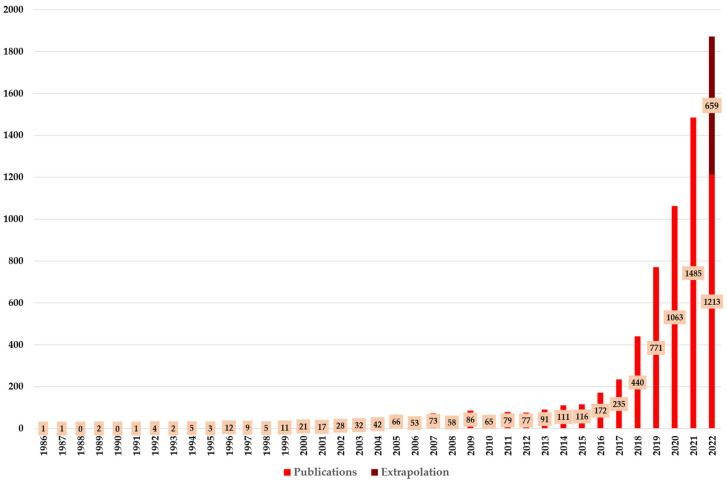
Overview of the annual production.

Figure 2 shows the annual production of research dealing with AI for cancer detection. The first research dealing with that topic was published in the 1980s. The first article can be dated to 1986. In this article, an expert system for the early detection of cervical cancer was proposed [60]. Until 1995, AI for cancer detection only experienced small growth in terms of annual production. In 1988 and 1990, no articles on this topic were published at all. In the following years, the number of publications only grew slowly. With 111 publications, the annual productions first topped the hundred mark in 2014. As the importance and potential of AI in general have increased, so has AI gained relevance in the field of cancer detection. As a result, most publications have been published in recent years (2019–2021). In 2022, 1213 publications had already been published before we collected the data for our study (23 September). Since it appears like statistically more publications are published in the last months of a year [21], we assume that the trend of increasing publications will be ongoing in 2022. Based on an extrapolation, we assume the total number of publications for 2022 will be 1872, with an estimate of 659 articles published after 23 September.

**Figure 3:**
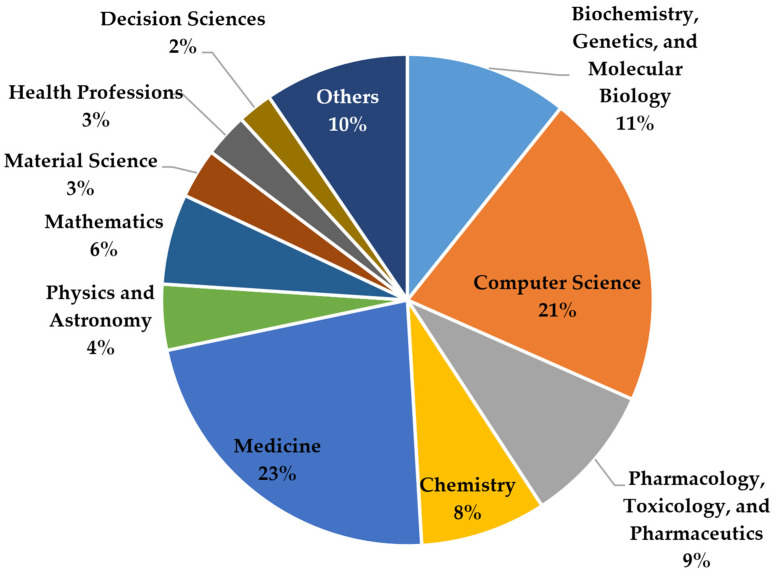
Overview of the most contributing disciplines.

Figure 3 displays the distribution of disciplines among the publications. The data of Figure 3 were derived from Scopus wherein a publication is assigned to a discipline based on the outlet it was published in. However, some journals or conferences can belong to more than one discipline. Not surprisingly, we see that medicine and computer science outlets are the most popular ones within AI for cancer detection. A total of 23% and 21% of all articles have been published in outlets that belong to these disciplines. Medicine and computer science are followed by biochemistry, genetics, and molecular biology (11%); pharmacology, toxicology, and pharmaceutics (9%); and chemistry (8%). The dominance of medicine and computer science is not surprising, since oncology and the detection and treatment of cancer is one of the central disciplines in medicine, while AI is traditionally rooted within computer science.

### 4.2. Sources, Countries, and Affiliations

In this subsection, we show the results of our performance analysis, wherein we focus on the contributions of different research constituents. First, we take a look at the most relevant sources in terms of their absolute publication count within our sample. Table 3 shows the 20 sources with the most publications on AI for cancer detection. In total, the 6450 articles of our sample were published in 2018 different sources, which is equal to 3.2 publications per source. With 169 publications, Lecture Notes in Computer Science is the most important outlet in terms of absolute publication count. Lecture Notes in Computer Science is followed by Progress In Biomedical Optics And Imaging Proceedings Of SPIE (110 publications), Cancers (94 publications), and Computers In Biology And Medicine (88 publications).

In Table 4, we show the 20 most productive countries within AI for cancer detection in terms of absolute publication count. An article is assigned to a county when one of its authors is affiliated with one institution or company that is located within that country. Due to international collaboration, one article can therefore be assigned to more than one country. Hence, the total number of articles in Table 4 exceeds the total number of publications within our sample. Next to the total number of published articles, we also show the average age of the documents, as well as the average number of citations each document has received. Additionally, Table 4 shows the percentage of international co-authorship for every country. For example, an international co-authorship percentage of 50% would mean that 50% of the articles of one country have at least one author of another country.

In total, authors from 118 countries have contributed to research on AI for cancer detection. This very high number of contributing countries underlines the global importance of this topic. With 1627 articles, authors from the United States were the most productive ones. The United States are followed by China with 1202 contributions and India (1079 publications). The United Kingdom follows with a large gap (411 articles), Canada (264 publications) is in the fifth place. With 262 articles, the first European country to appear in the list is Germany in the sixth rank. Next to Germany, four other countries of the European Union are among the 20 most contributing nations, namely Italy, Spain, the Netherlands, and France.

When we look at the average age of the articles, it is striking that the United States not only has the most articles but also the oldest ones. In average, contributions from the United States have an age of 4.76 years. This is more than one year above the average age of the total sample. Among the top 20 countries, only Germany (4.6 years), Taiwan (4.33 years), Iran (4.1 years), and Spain (4.06 years) have an average article age of more than 4 years. This shows that these five countries are traditional contributors within the field of AI for cancer detection. It is striking that the contributions of China, the country with the second-most publications within our sample, are significantly younger. In average, Chinese contributions were 2.22 years old. This indicates that Chinese authors have contributed a lot, especially in the last few years. Only Saudi Arabia (1.24 years) and Pakistan (1.69 years) have younger articles on average. 

Additionally, Table 4 shows the average citations the publications from a given country have received. The highest average citation numbers can be found for articles authored by authors from France (55.95 citations), Germany (47.50 citations), and the Netherlands (41.17 citations). The United States has received 36.32 citations on average, and Chinese publications have received 16.17. However, large parts of the different average citation counts can be explained with the average age of the articles. The average number of citations per document correlates with the average age of the articles, since recent articles have not had time to receive a high number of citations [47,61]. Additionally, it is interesting to observe that Indian articles received a much lower number of citations on average than Chinese ones (9.179 vs. 16.17), although the average age of the publications is relatively close to each other. However, it might be possible to explain this by the percentage of international co-authorship. While China has an international co-authorship ratio of 32.36%, this value is significantly lower for India (16.70%). Given that, it can be assumed that Indian research is much more isolated and probably not so much known in other countries, leading to a lower citation score.

The highest ratios of international co-authorship can be found for articles authored or co-authored by researchers from Saudi Arabia (74.64%), Australia (70.47%), Pakistan (67.42%), and the United Kingdom (66.35%). The lowest scores can be found for Indian (16.70%), Iranian (30.83%), Turkish (31.62%), and Chinese (32.36%) contributions. Furthermore, we can see that the average percentage of international co-authorship of the 20 most contributing countries is much higher than this value for the whole sample (24.97%). Likewise, this shows that many countries with only a few contributions tend to have a comparatively low amount of international co-authorship. 

**Figure 4:**
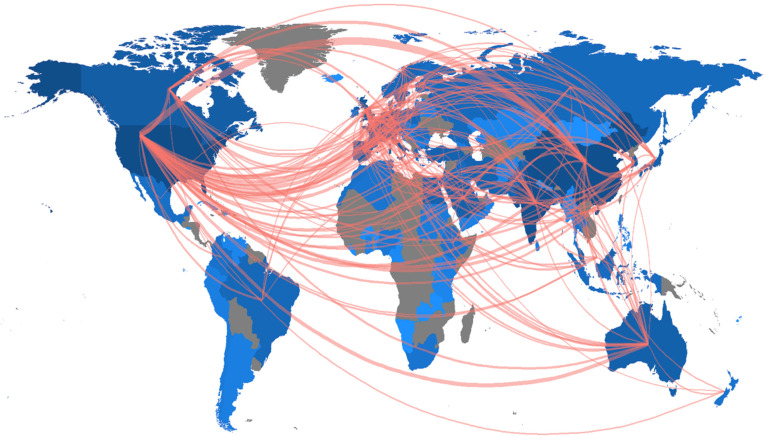
International co-collaboration map (generated with Biblioshiny).

To further illustrate the international collaboration, Figure 4 shows an international collaboration map. Herein, collaborations between different countries are depicted with red lines. The thicker a red line between two countries is, the more collaboration took place among researchers of these two nations. To not overload it, only relationships with at least three contributions between two countries are depicted in Figure 4. Additionally, the countries’ color represents their number of publications. The darker the blue is, the more publications have been contributed from researchers a specific country. Herein, we can see three large centers of collaboration, namely in the United States, China, and the European Union. These three areas have a lot of different collaborations with many different countries.

Table 5 presents the 20 institutions and organizations that funded the most articles. With 539 publications, the National Natural Science Foundation of China has funded the most articles on AI for cancer detection. It is followed by the National Institutes of Health (408 publications), the National Cancer Institute (336 publications), and the National Science Foundation (113 publications). It is noteworthy that the top three funding sponsors together funded 1283 articles, which is almost equivalent to 20% of all publications dealing with AI for cancer detection. Both China and USA are most often represented, each with six funding sponsors among the top 20. They are followed by the European Union with three and Canada and UK with two funding sponsors.

Finally, Table 6 shows the 20 affiliations that authored the most publications within the field of AI for cancer detection. An article is assigned to one affiliation based on the contributing authors. Since an article can be authored or co-authored by researchers from different institutions, certain articles can be linked to more than one affiliation. With 219 articles, researchers from Sichuan University contributed to the most publications dealing with AI for cancer detection. The Sichuan University is followed by three affiliations located in the United States, namely the University of California (199 publications), the Memorial Sloan Kettering Cancer Center (195 publications), and the Stanford University (170 publications). Of the 20 most contributing affiliations, eight are located in China and eight in the United States. Additionally, one affiliation is from the Netherlands (Radboud University Medical Center, 145 publications), Japan (The University Of Tokyo, 95 publications), the United Kingdom (University Of Cambridge, 90 publications), and Canada (University Of Toronto, 90 publications).

### 4.3. Content Analysis

In this section, we will thematically dive into the topics that are dealt with in AI for cancer detection research. First, Table 7 shows the 25 most frequently used keywords in our sample. This does not only include author keywords but also indexed keywords from Scopus. The keywords “human” and “humans” were most-often used, which indicates that most of the research belong to human medicine, specifically, cancer that affects humans. This is followed by “cancer diagnosis” and “diseases”. The most frequently used technical keywords and terms in our sample were “deep learning” (2275 appearances), “machine learning” (2163 appearances), and “artificial intelligence”, which appeared 1735 times. Other frequently used technologies according to the most often used keywords are “convolutional neural networks” (1021 appearances) and “artificial neural networks” (903 appearances).

When we specifically focus on cancer types, breast cancer is most frequently addressed in the articles dealing with AI for cancer detection. With 1498 appearances, breast cancer is the ninth of the most often-used keywords. This is not surprising, since breast cancer is the most common carcinoma among women globally and comes with a low survival rate [62]. Breast cancer is followed by lung cancer (598 appearances), which causes the most cancer-related deaths worldwide [63]. Breast and lung cancer are followed by prostate cancer (425 appearances) and melanoma (skin cancer, 247 appearances).

**Figure 5:**
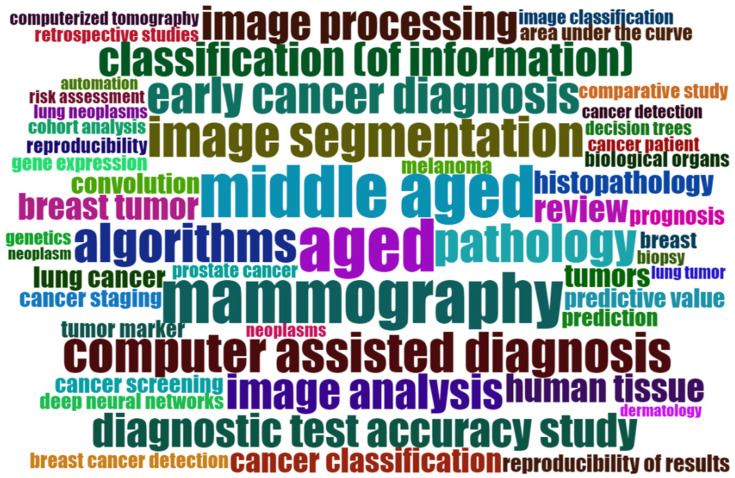
Word cloud of the most-frequently appearing keywords plus (generated with Biblioshiny).

To obtain a deeper understanding of the topics dealt with, Figure 5 shows a word cloud of the most frequently used keywords plus. Keywords plus are another way to analyze a document’s content and are automatically generated out of words or phrases that are frequently used in the titles of an article’s references [64,65]. In Figure 5, the size of words is determined based on their frequency in the keywords plus. Herein, many of the most frequently identified words are closely related to the cancer types that are most often addressed (e.g., “mammography”, “lung cancer”, “breast tumor”, or “melanoma”), which is not a surprising result.

**Figure 6:**
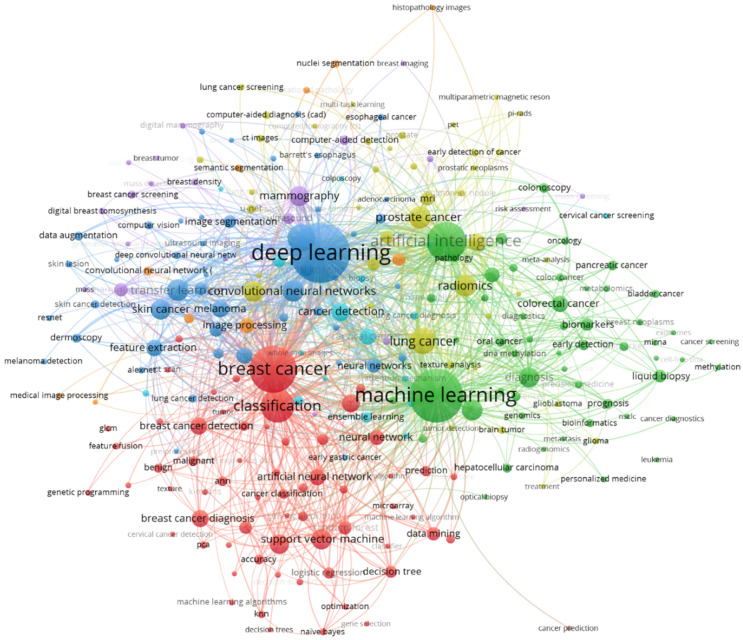
Network of keyword co-occurences.

Additionally, Figure 6 shows a keyword co-occurrence network of author keywords and indexed keywords of our sample. Like in the word cloud in Figure 5, the font size depends on the frequency a term is used. Terms that frequently appear together are linked with lines and are arranged in clusters of the same color. Terms that appear in the center of the network, such as “deep learning”; “machine learning”, “artificial intelligence”, or “machine learning”, are connected with many other words in the network. It is noteworthy that it is hard to distinguish clear thematical clusters based on the color in Figure 6. Although a red and a green cluster are visible, the keywords that belong to these clusters have many relations to terms that do not belong to these clusters. Keywords in yellow, blue, or purple, for example, are spread in the whole network and to not represent clearly distinguishable thematic fields. Despite the fact that AI for cancer detection is a multidisciplinary field, we can conclude from Figure 6 that knowledge and research on that topic is not fragmented. Although different clusters can be identified, these are not isolated from other research streams, which shows the overall coherence within that research field.

Finally, Table 8 shows the 30 most-cited articles in our sample. A total of 13 of the 30 articles do have a general focus on AI’s potential for drug discovery and do not focus on a single cancer type. Among the other articles, breast cancer (10 publications) is the cancer type that is most often addressed, followed by brain tumors (3 publications). With 2136 citations at the point of time our data were collected, the article “Classification and diagnostic prediction of cancers using gene expression profiling and artificial neural networks” is the most often cited publications in our sample. In their article, the authors show the potential and applications of artificial neural networks for diagnosing cancer and the identification of candidate targets for therapy. Although this article is comparatively old and has been published in 2001, the results were already promising and showed the great potential of artificial neural networks. In rank two, the article “The evaluation of tumor-infiltrating lymphocytes (TILs) in breast cancer: recommendations by an International TILs Working Group 2014” follows with 1533 citations. Although AI and ML is only partly covered in this article, the authors mention ML to be a promising tool for the future assessment of TILs [66] (p. 269). 

## 5. Future Research Agenda

In the prior sections, we presented the results of our bibliometric study. Based on our findings, we will present promising avenues for future research in this section. These have the purpose to serve as an orientation for interested scholars. 

First, considering the word cloud in Figure 5 and the focus of the most-cited studies, it becomes evident that the current state of research mainly focuses on the predictive performance of a limited number of applied AI algorithms. The interaction between the computer system and the humans involved, also referred to as human–computer interaction, is a topic addressed much more rarely. It is important to investigate how the interaction between AI and the humans may or should look in the context of cancer diagnosis. In general, there are different conceivable scenarios, namely substitution, augmentation, and assemblage [95,96]. Augmentation refers to the scenario that AI and humans augment each other, while assemblage means that the AI and humans are brought together dynamically to function as a unit. Finally, substitution means that the human is completely replaced by the AI system [96]. Future research needs to investigate which form of cooperation between AI and humans is most suitable in the context of cancer diagnosis. This involves the question of whether a substitution is possible and, especially, if it is desirable, at all. There are already a few promising studies available that investigate human–computer interaction in the health industry [97,98]. Therefore, these studies can be used as a foundation for future studies that address the relationship between AI and humans. Additionally, trust between the AI cancer detection model and humans involved is an important factor. Although AI systems often have accuracy that surpasses that of human experts, there is a lack of trust in the predictions generated by AI systems [99]. It should be therefore investigated what reasons exist for a lack of trust and how trust in the AI system can be improved. This also holds true for patients who might be subject to treatments that are mainly based on the results of an AI system. Explainable artificial intelligence (XAI, see below) might be one way to increase the trust in an AI system. 

One important aspect is also the security and robustness of the AI models. Many AI models that are described in the literature were evaluated only on one dataset. Therefore, it might remain unclear if the AI model can be transferred to input data that stems from different scanning machines. Therefore, it would be worthwhile to investigate how AI models must be designed to ensure their transferability [100,101,102]. In this context, it also might make sense to evaluate AI models using several datasets generated by different sensors or different manufacturers. As outlined above, AI systems require a large amount of data to learn and to develop robust models. When it comes to data, it is additionally important to ensure the trustworthiness, reliability, and security of the sources or platforms the data stem from [103,104]. If malicious actors succeed in manipulating or changing the data that are used as an input for the AI system, this might affect the AI system’s result. Therefore, these results are not reliable anymore and might endanger the patient’s health due to the risk of wrong results. Data storage is an especially important aspect, as medical data is subject to special data-protection regulations. Therefore, it should be examined what storage solutions are compliant with regulations, such as the GDPR or HIPAA, and how to ensure that the data is not traceable. In this context, future research should also verify whether the pseudonymization of the data is sufficient or whether complete anonymization is required. Different researchers also examine whether new technologies for the distributed storage and management of data, such as the blockchain, might be suitable for medical data [105,106,107]. Future research could therefore take a critical look if a blockchain would make sense for the purpose of managing and storing medical data or if other technologies and databases are more suitable. Moreover, it is noteworthy that there are already a few studies available that investigate security and robustness aspects of AI models for cancer detection. Approaches such as the external validation of AI algorithms [108] and robustness tests against adversarial images [109], as well as comprehensive data preprocessing [110,111], are promising to achieve robustness and security goals and should therefore be investigated in more detail. In this context, the application of design science research could also be a way to iteratively address specific security problems in order to find an efficient solution. Examples of design science research can be found in business administration [31,112] and information systems [113,114]. 

As mentioned above, the explainability of an AI model is an important factor to ensure the acceptance for and trust in an AI model. With an AI system’s advancing complexity, it is increasingly difficult to understand how it comes up with its results and predictions. This holds true for the most of today’s AI and machine learning algorithms, which are very complex and [21,115] considered black boxes. Although XAI is hard to achieve, it is necessary for certain use-cases in critical areas like law or medicine [116,117,118]. For cancer detection, XAI can be considered very relevant. This might not be the case as long as the AI system’s results are doublechecked by doctors or oncologist. However, before AI can be used independently, explainability is an important challenge that needs to be addressed [119]. Recent reviews and surveys demonstrate that XAI in medicine is still one of the most signifcant research gaps and remains largely unanswered [102,120]. Future research should therefore investigate how AI systems for cancer detection can be made transparent enough that their results are understandable. It might make sense to collaborate with AI researchers or scholars from other disciplines since some promising XAI applications and developments might not yet be applied in the context of cancer detection.

Table 9 below provides an overview of our future research agenda and presents possible future research questions that might help advance the field of AI for cancer detection.

## 6. Discussion

In this study, we conducted a bibliometric analysis of 6450 articles dealing with the potential and application of AI for cancer detection and diagnosis. To the best of our knowledge, this is the first study that uses a bibliometric approach to analyze research on AI for cancer detection. This study has several implications and benefits for both researchers and practitioners. First, interested researchers can use the study at hand to obtain an initial overview of research on AI for cancer diagnosis. This involves information about the scientific landscape and the most influential articles, as well as core topics and key themes investigated. As such, this study can help to equip interested scholars with an initial understanding of the research field dealing with AI-based cancer detection. Our research agenda furthermore can serve as a foundation for future research to build on to further develop this exciting field. Additionally, both clinical as well as commercial practitioners can use our study to obtain an initial insight about the potential of AI for supporting the diagnosis of cancer.

Our study is subject to certain limitations. First, we used Scopus as the only scientific database for the collection of our bibliometric data. Although, as outlined above, Scopus covers a huge number of different conferences and journals, it is likely that different publications were not covered by our research. Bibliometric studies on AI for cancer detection that use other databases for data collection might therefore lead to slightly different results. However, we believe that the most of our key results, especially the most important topics and key themes, are likely to maintain constant even if other databases would be used. Furthermore, the application of other bibliometric tools and analysis methods like citation analysis [121] or bibliographic coupling [122,123] might lead to additional results that were not part of this study. Additionally, AI is a fast-evolving field. New research on AI for the purpose of cancer detection is published every month. This study’s results are therefore only able to show the current state of the art. 

## 7. Conclusions

AI is a promising technology that is increasingly applied to detect or diagnose cancer. In recent years, research on AI for cancer detection grew rapidly, resulting in a high number of research articles on that topic. Due to the large amount of research that is available, it is hard for interested scholars or clinical practitioners to obtain an initial understanding of this field. Against this backdrop, we aimed to provide researchers with an overview and analysis of the research field of AI for cancer detection. For this purpose, we conducted a bibliometric study of the existing research on that topic. In total, we identified and analyzed 6450 articles published between 1986 and 2022. 

Our analysis consisted of different parts. First, we gave a general overview of our sample and presented the development of scientific production over the year and which disciplines contributed to it. After that, we conducted a performance analysis. Herein, we identified the most productive institutions and countries. Additionally, we gave an overview of the most relevant outlets and the international collaboration. Finally, we thematically analyzed the sample and identified key topics and the most-cited publications. We found that breast and lung cancer are cancer types most often addressed by recent research. Based on these findings, we developed a future research agenda that is supposed to guide researchers to further advance the field of AI-based cancer diagnosis. We believe that we provide a systematic and holistic overview of this exciting field of research and hope that our study will serve interested scholars and practitioners as a valuable overview.

## Figures and Tables

**Table 1:** Main information and general metrics.

Metric	Value
**Main information**	
Timespan of publications	1986–2022
Sources (conferences and journals)	2018
Documents	6450
Average citations per document	19.87
Average document age	3.72
Total number of references	247,762
Number of author’s keywords	9321
Number of keywords plus	21,192
**Document types**	
Journal article	4016
Conference article	1729
Review	708
**Authors and collaboration**	
Number of different AI-cancer authors	23,854
Documents per AI-cancer author	0.270
Single-authored documents	218
Multi-authored documents	6232
Authors of multi-authored documents	23,651
Co-authors per document	5.89
Collaboration index	3.8
International co-authorship	24.97%

**Table 2:** Comparison of different bibliometric studies.

Study	[58]	[59]	[21]	[48]	This Study
Topic	Data quality	Blockchain in accounting	AI for drug discovery	Data governance	AI for cancer detection
Documents	159	93	3884	780	6450
Documents per author	0.305	0.443	0.322	0.367	0.27
Collaboration index	3.60	2.83	3.26	3.26	3.8
Single-authored documents	-	29%	6.7%	22.18%	3.4%

**Table 3:** Overview of the sources with the most publications.

Rank	Source	Publications
01	Lecture Notes in Computer Science	169
02	Progress In Biomedical Optics and Imaging Proceedings Of SPIE	110
03	Cancers	94
04	Computers in Biology and Medicine	88
05	Computer Methods and Programs in Biomedicine	81
06	Scientific Reports	79
07	Plos One	71
08	European Radiology	69
09	Diagnostics	68
10	IEEE Access	64
11	Artificial Intelligence in Medicine	61
12	Medical Image Analysis	59
13	Proceedings Of SPIE The International Society for Optical Engineering	58
14	Frontiers in Oncology	55
15	Medical Physics	55
16	IEEE Transactions on Medical Imaging	53
17	Advances in Intelligent Systems and Computing	50
18	Computerized Medical Imaging and Graphics	47
19	Biomedical Signal Processing and Control	46
20	ACM International Conference Proceeding Series	45

**Table 4:** Overview of the countries with the most publications.

Rank	Country	Articles	Avg. Age (Years)	Avg. Cit.	Int. Co-Authorship
01	United States	1627	4.76	36.32	44.26%
02	China	1202	2.22	16.17	32.36%
03	India	1079	2.39	9.179	16.70%
04	United Kingdom	411	3.9	33.05	66.35%
05	Canada	264	3.52	33.94	55.88%
06	Germany	262	4.6	47.50	59.33%
07	Italy	248	3.73	28.39	55.21%
08	South Korea	221	2.53	23.63	40.95%
09	Japan	208	3.35	29.21	36.70%
10	Saudi Arabia	196	1.24	10.53	74.64%
11	Australia	190	3.36	32.81	70.47%
12	Spain	178	4.06	34.12	57.22%
13	Netherlands	177	2.8	41.17	64.90%
14	France	165	3.44	55.95	63.10%
15	Egypt	144	2.33	15.53	44.67%
16	Turkey	137	3.57	39.62	31.62%
17	Malaysia	134	3.36	17.31	49.50%
18	Iran	131	4.1	15.85	30.83%
19	Pakistan	123	1.69	13.78	67.42%
20	Taiwan	119	4.33	28.26	39.34%

**Table 5:** Overview of the funding sponsors with the most funded publications.

Rank	Funding Sponsor	Country/Region	Quantity
01	National Natural Science Foundation of China	China	539
02	National Institutes of Health	USA	408
03	National Cancer Institute	USA	336
04	National Science Foundation	USA	113
05	National Key Research and Development Program of Chinas	China	106
06	U.S. Department of Health and Human Services	USA	89
07	Fundamental Research Funds for the Central Universities	China	79
08	National Research Foundation of Korea	South Korea	67
09	Natural Sciences and Engineering Research Council of Canada	Canada	60
10	European Regional Development Fund	EU	58
11	European Commission	EU	57
12	National Institute of Biomedical Imaging and Bioengineering	USA	50
13	Japan Society for the Promotion of Science	Japan	48
14	Ministry of Education of the People’s Republic of China	China	40
15	Ministry of Science and Technology of the People’s Republic of China	China	40
16	Canadian Institutes of Health Research	Canada	39
17	Cancer Research UK	UK	36
18	Science and Technology Commission of Shanghai Municipality	China	36
18	National Institute for Health Research	UK	34
19	Horizon 2020 Framework Programme	EU	33
20	Nvidia	USA	32

**Table 6:** Overview of the affiliations with the most publications.

Rank	Affiliation	Country/Region	Articles
01	Sichuan University	China	219
02	University of California	USA	199
03	Memorial Sloan Kettering Cancer Center	USA	195
04	Stanford University	USA	170
05	Fudan University	China	165
06	Shanghai Jiao Tong University	China	151
07	Harvard Medical School	USA	147
08	Huazhong University of Science and Technology	China	145
09	Radboud University Medical Center	Netherlands	145
10	University of Pennsylvania	USA	132
11	Southern Medical University	China	130
12	National Cancer Institute	USA	107
13	University of British Columbia	USA	104
14	Renmin Hospital of Wuhan University	China	101
15	Zhejiang University	China	101
16	The University of Tokyo	Japan	95
17	Emory University	USA	94
18	Sun Yat-Sen University Cancer Center	China	93
19	University of Cambridge	UK	90
20	University of Toronto	Canada	90

**Table 7:** Overview of most frequently used keywords.

Rank	Keyword	Quantity
01	Human	3585
02	Humans	2685
03	Cancer Diagnosis	2621
04	Diseases	2521
05	Deep Learning	2275
06	Machine Learning	2163
07	Artificial Intelligence	1735
08	Female	1648
09	Breast Cancer	1498
10	Sensitivity and Specificity	1407
11	Controlled Study	1336
12	Diagnosis	1325
13	Diagnostic Accuracy	1273
14	Diagnostic Imaging	1245
15	Major Clinical Study	1199
16	Procedures	1123
17	Male	1092
18	Priority Journal	1088
19	Medical Imaging	1081
20	Adult	1061
21	Convolutional Neural Network	1021
22	Algorithm	1016
23	Computer Aided Diagnosis	909
24	Artificial Neural Network	903
25	Learning Systems	882

**Table 8:** Overview of the 30 most-often cited articles.

Rank	Authors	Year	Focus	Citations	Reference
01	Khan et al.	2001	General investigation	2136	[67]
02	Salgado et al.	2015	Breast cancer	1533	[66]
03	Kourou et al.	2015	General investigation	1426	[68]
04	Bejnordi et al.	2017	Breast cancer/lymph node metastases	1305	[69]
05	Lu and Fei	2014	General investigation	1252	[70]
06	Coudray et al.	2018	Lung cancer	1018	[71]
07	McKinney et al.	2020	Breast cancer	774	[72]
08	Johnson et al.	2019	General investigation	720	[73]
09	Cruz and Wishart	2006	General investigation	693	[74]
10	Statnikov et al.	2005	General investigation	644	[75]
11	Spanhol et al.	2016	Breast cancer	626	[76]
12	Haenssle et al.	2018	Skin cancer	588	[77]
13	Litjens et al.	2016	Breast cancer/prostate cancer	581	[78]
14	Mazurowski et al.	2008	Breast cancer	557	[79]
15	Akay	2009	Breast cancer	554	[80]
16	Bi et al.	2019	General investigation	553	[81]
17	Zacharaki et al.	2009	Brain tumors	542	[82]
18	Shrestha and Mahmood	2019	General investigation	525	[83]
19	Tang et al.	2009	Breast cancer	488	[84]
20	Statnikov et al.	2008	General investigation	467	[85]
21	Irshad et al.	2014	General investigation	450	[86]
22	Zhao et al.	2018	Brain tumors	428	[87]
23	Dou et al.	2017	General investigation	421	[88]
24	Zheng et al.	2014	Breast cancer	374	[89]
25	Lee et al.	2008	General investigation	372	[90]
26	Limkin et al.	2017	General investigation	364	[91]
27	Albarqouni et al.	2016	Breast cancer	360	[92]
28	Urban et al.	2018	Polyps/Colorectal cancer	347	[16]
29	Ribli et al.	2018	Breast cancer	346	[93]
30	Işın et al.	2016	Brain tumor	345	[94]

**Table 9:** Future research agenda.

Focus	Possible Research Questions
Human Computer Interaction	How can the interaction between doctors and AI models be designed efficiently?
	What is the current state of trust towards AI based models in medicine?
	How can trust in AI be built for doctors and patients?
	How can AI experts and clinical practitioners cooperate and work together in the best way?
	What is the role of explainable AI for building trust?
Robustness and security	How reliable are trained AI models on other cancer datasets (e.g., generated by other sensors)?
	Can adversarial attacks outsmart AI models in medicine?
	How should an AI system for cancer detection be designed to make it robust and secure against adversarial attacks and actors?
	Could a cancer detection algorithm be applied to other types of cancer?
Explainable AI	Should explainable AI models be preferred instead of the most accurate one?
	How should explainable AI be designed to increase the trust in the AI system and its decisions?
	What are the promising approaches in XAI that have not yet been applied in the medical field?
Data Storage	Where should the data of the scans be stored to ensure data privacy rights?
	Do new technologies like blockchain have a potential for the storage and management of medical data?
	Should patient data be irreversibly anonymized or only pseudonymized?

## Data Availability

Not applicable.
